# An Integrative Network Analysis Framework for Identifying Altered Glycosylation Pathways Associated with Autism Spectrum Disorder

**DOI:** 10.3390/genes17040486

**Published:** 2026-04-19

**Authors:** Anup Mammen Oommen, Marie Morel, Stephen Cunningham, Cathal Seoighe, Lokesh Joshi

**Affiliations:** 1 Glycoscience Research Cluster (AGRC), University of Galway, Biomedical Sciences, H91 W2TY Galway, Ireland; anupmammen.oommen@universityofgalway.ie (A.M.O.); stephen.cunningham@universityofgalway.ie (S.C.); 2 Institute for Health Discovery & Innovation, University of Galway, H91 TK33 Galway, Ireland; 3 UMR 8576-UGSF-Unité de Glycobiologie Structurale et Fonctionnelle, Univ. Lille, CNRS, F-59000 Lille, France; marie.morel@univ-lille.fr; 4 School of Mathematical and Statistical Sciences, University of Galway, H91 TK33 Galway, Ireland; cathal.seoighe@universityofgalway.ie

**Keywords:** molecular function, glycosylation, inflammatory pathways, neurological functions, network analysis, complex disorders, neurobehavioral symptoms, variant-gene association, LD analysis, eQTLs

## Abstract

**Background**: Autism Spectrum Disorder (ASD) is a complex neurodevelopmental condition marked by heterogeneous behavioral symptoms and systemic comorbidities, including immune and gastrointestinal dysfunctions. Emerging studies suggest that glycosylation—a fundamental post-translational modification regulating cellular communication and immune responses—may play a role in ASD pathophysiology, yet its contribution remains underexplored. **Methods**: In this study, we developed an integrative transcriptomic and network analysis framework to investigate glycosylation-related gene expression changes and their functional associations in ASD. Using publicly available datasets from bulk and single-cell RNA sequencing of brain and blood tissues, we focused on four prior-knowledge gene subsets: glycogenes, extracellular matrix glycoproteins, immune response genes, and autism risk genes. **Results**: Differential expression and pathway enrichment analyses revealed consistent dysregulation of glycosylation pathways, including mucin-type *O*-glycan biosynthesis, glycosaminoglycan metabolism, GPI-anchor formation, and sialylation, across ASD tissues. These transcriptional changes were functionally linked to altered immune signaling (e.g., IL-17, Toll-like receptor, and complement pathways) and synaptic development pathways, forming a distinct glyco-immune axis. Network analysis identified key glycogenes such as *GALNT10*, *NEU1*, *LMAN2L*, and *CHST1* as central molecular nodes, interacting with immune and neuronal regulators. Linkage disequilibrium analysis further revealed ASD-associated SNPs influencing the expression of these glycogenes in both blood and brain tissues. **Conclusions**: Together, these findings support a model in which disrupted glycosylation contributes to ASD pathophysiology by mediating immune dysregulation and altered neuronal connectivity. This study offers a systems-level framework to understand the molecular complexity of ASD and highlights glycogenes as potential biomarkers and targets for future therapeutic exploration.

## 1. Introduction

Autism spectrum disorder (ASD) is currently recognized as a complex neurodevelopmental condition characterized by persistent deficits in social communication and interaction, alongside restricted, repetitive patterns of behavior, interests, or activities. (American Psychiatric Association, 1994) [1]. Current diagnostic frameworks, including the Diagnostic and Statistical Manual of Mental Disorders, Fifth Edition (DSM-5), and the International Classification of Diseases (ICD-11), define ASD as a spectrum condition, encompassing a wide range of clinical presentations [2]. This clinical heterogeneity—spanning variability in cognitive function, language ability, and associated comorbidities such as epilepsy and intellectual disability—poses a significant challenge to understanding its underlying biological mechanisms [3]. Both genetic and environmental factors contribute to ASD etiology. The genetic influences on the etiology of ASD have been evaluated through family-based ASD twin cohort studies [4], while population-based and molecular studies highlight the role of environmental influences and epigenetic modifications across brain and peripheral tissues [5,6]. In addition, recent studies have also emphasized the role of gut microbiota-derived metabolites in modulating epigenetic mechanisms, further contributing to ASD pathophysiology [7]. Consequently, ASD is considered a highly heritable and multifactorial disorder involving complex gene–environment interactions [8].

The rising prevalence of ASD reflects not only improvements in diagnostic practices but also potentially a true increase in incidence, driven by multiple interrelated factors [9]. The multilevel healthcare needs of ASD subjects owing to the endophenotypic complexity highlight the importance of systems-level understanding of the biological mechanisms affecting the quality of life (QoL) in ASD subjects [10]. The comorbid cluster in ASD largely comprises psychiatric disorders such as anxiety, attention-deficit/hyperactivity disorder, and intellectual disability, along with other systemic dysregulations such as gastrointestinal, immune system, and sleep disorders [11]. Currently, many areas, including genetics, epigenetics, immunology, microbiology and biochemistry, engaged in the ASD research, have unveiled the biological complexity predicting hundreds of candidate gene-disease association markers [5]. However, given the variability in the pattern and severity of symptoms in ASD, a consistent association of these markers with the behavioral symptoms and comorbidities among the ASD subjects needs to be established. In this regard, despite the challenges in the identification of genetic variants (such as single-nucleotide polymorphisms (SNPs) and copy number variations (CNVs), significant advancements have been made in clinical and laboratory human genetic research, resulting in the identification of ASD risk genes. Databases such as Simons Foundation Autism Research Initiative (SFARI) genes (https://gene-archive.sfari.org/) are still contributing to the classification and assessment of these susceptibility genes as syndromic and non-syndromic based on clinical evidence, thus laying the foundation for the development of predictive genetic tests in the near future [12].

Interestingly, several variants reported in SFARI genes (https://gene.sfari.org/database/gene-scoring/, accessed on 2 October 2020) occur in genes that encode for glycoconjugates such as proteoglycans or glycoproteins, glycosphingolipids, enzymes involved in glycosylation (e.g., glycosyltransferases, glycosidases and sulfotransferases), as well as glycan-binding proteins, collectively referred to as “glycogenes”. Abnormal regulation of glycosylation pathways has been previously implicated to be correlated with some of the most common comorbidities in ASD [13], such as epilepsy [14], psychiatric/behavioral complaints [15,16], as well as gastrointestinal (GI) disorders [17,18]. Glycosylation is indeed a key post-translational modification (PTM) of proteins and lipids. The resultant glycoconjugate structures are the critical mediators of the major biological processes involved in brain development [19], immune response [20,21], as well as in core gastrointestinal functions, which are significantly affected in the above-mentioned comorbidities [22]. Congenital disorders of glycosylation (CDG) are a genetically and clinically heterogeneous group of over 190 rare metabolic diseases caused by defects in various steps of glycosylation pathways. Initial observations linking altered glycan patterns with autistic behavior have been observed in CDG patients [14] and have led to the identification of major glycogenes associated with idiopathic, syndromic and familial ASDs [23]. On this note, alteration in serum α2-3 sialoglycosylation as well as elevated GM1-type glycosaminoglycan excretion in the urine samples of autistic children have been reported [24,25]. Moreover, in 2011, a study by Pivac N et al. reported an absence of changes in plasma *N*-glycome in association with ASD phenotype compared to attention-deficit hyperactivity disorder phenotypes [15]. Further, a genome-network analysis study conducted by van der Zwaag B et al. in 2009, has identified genomic gain or loss in 7 major gene-coding glycoenzymes as *B3GALT6*, *GNCT2* and *GALNT9*, which are proposed as important contributors to the development of ASD [26]. Numerous factors regulate the complex mammalian glycome abundance on biological macromolecules and the transcriptional control of the glycogenes is hypothesized to determine the glycan abundance and diversity [27].

Thus, the continued generation of large and publicly available ‘omic’ data resources presents an opportunity to examine the biological relevance of the altered expression of glycogenes in complex disorders like ASD through a tiered systems biology approach to identify novel biological and clinical relationships. However, most of these approaches focus on transcriptomic data-driven pathway enrichment analysis methods, which often miss the importance of non-genetic molecular measurements due to the gap in gene coverage representing post-translational modifications (PTM) in established enrichment analysis databases. Recently, we published an integrative analysis framework for the detailed biological process enrichment analysis associated with the Major Depressive Disorder (MDD) phenotype to identify the biological role and significance of glycosylation PTM [25,28]. A similar approach was leveraged in the current study to compile a comprehensive coverage of transcriptional alteration of glycogenes in autistic subjects in comparison with the extracellular matrix (ECM) glycoproteins, inflammatory genes and autism risk genes to assess their correlation with the altered neurobehavioral symptoms and other comorbidities by leveraging publicly available gene expression datasets.

## 2. Materials and Methods

The primary goal was to evaluate the functional significance and potential of this methodology in the identification of molecular function and biological pathway association of transcriptionally altered glycogenes in ASD and its correlation with the biological pathways represented by altered expression patterns of inflammatory, ECM glycoproteins and autism risk genes. The design methodology explored for the integrative analysis encompassing transcriptomic assessment, reported bibliographic databases, open knowledge platforms and ‘omic’ databases is depicted in Supplementary Figure S1.

### 2.1. Gene Expression Data Selection and Processing

For the transcriptomic analysis, the public database Gene Expression Omnibus (GEO) (https://www.ncbi.nlm.nih.gov/geo/ accessed on 2 October 2020) was queried using the keyword “Autism Spectrum Disorder”. Datasets included both blood and brain samples from ASD subjects and only those datasets with ≥3 replicates for both test and control samples were considered. Datasets without gene annotation were excluded from the analysis. For data processing and gene annotation, online software tools such as GenePattern version 2.0 (http://software.broadinstitute.org/cancer/software/genepattern/ accessed on 2 October 2020) [29] and Galaxy version 21.01 (https://usegalaxy.org/) [30] were used throughout, following the procedures detailed in user manuals. In GenePattern, preprocessing of RNA-Seq data, including normalization, missing value imputation, and collapsing multiple probe sets’ expression values into single expression values, was performed using VoomNormalize (v2); ImputeMissingValues and CollapseDataset modules, respectively. Differential expressions of the preprocessed data files were then performed for individual datasets using the ComparativeMarkerSelection module. While in Galaxy, the limma-voom tool was utilized for the differential expression analysis, accepting the default Trimmed Mean of M values (TMM) normalization method and applying filters to remove lowly expressed genes in each dataset selected for the analysis (we chose to retain genes if they are expressed at a count per million above 0.5 in at least three samples). The significance of marker genes was calculated using default *p*-value adjustment methods in the analysis. For a few datasets, normalized, log2-transformed gene expression matrix files were used directly from the GEO databases, wherever available. A data analysis tool in Microsoft Excel software was used for summarizing the descriptive statistics of the differentially expressed genes from the processed gene expression datasets.

### 2.2. Gene Sets Based on Prior Knowledge

For the gene expression analysis of glycosylation process-related genes, the GlycoGAIT database [31], an interactive web database developed within our research group, was utilized. The detailed methodology employed for enriching and developing the database was as described previously [28]. For the autism risk gene, we used the human gene module provided in the SFARI database (https://gene.sfari.org/tools/; downloaded in 2 October 2020). For generating a unique list of human gene markers relevant to immune response, we manually compiled the gene list from multiple data sources available from InnateDB (https://www.innatedb.com/redirect.do?go=resourcesGeneLists accessed on 2 October 2020), by querying for gene families and associated genes representing immune markers from the HUGO Gene Nomenclature Committee (HGNC) database (https://www.genenames.org/). Additional enrichment of gene markers was identified from GO database (http://geneontology.org/) by keying in 19 relevant query terms relevant to immune response such as “activation of immune response”; “antigen processing and presentation”; “complement-dependent cytotoxicity”; “cytokine production”; Immune; “Immune Response”; “immune system development”; “Immunoglobulin complex”; “inflammasome complex”; Inflammatory; “leukocyte activation”; “leukocyte homeostasis”; “leukocyte mediated cytotoxicity”; “leukocyte migration”; “leukocyte proliferation”; “lymphocyte costimulation”; “myeloid cell homeostasis”; “T cell selection”; “tolerance induction”. For compiling the unique list of ECM glycoproteins, we relied upon the human matrisome protein list available from the Matrisome Project, The Hynes Lab (http://matrisomeproject.mit.edu/ accessed on 2 October 2020).

### 2.3. Bibliographic Search and Gene Expression Data Selection

A systematic query of the PUBMED bibliographic database [25] using the search query (“autism spectrum disorder” [Title/Abstract]) AND (transcriptomic [Title/Abstract]) OR (“gene expression” [Title/Abstract]). Manual filtering of the data was adopted to select relevant transcriptomics studies that publish processed and/or raw data but are not reported in the public data repositories like GEO. We identified two blood-transcriptomic studies based on a discordant sibling pair design, as well as concordant and discordant monozygotic twin studies with autistic conditions by Domenici, E. et al. [32] and Meaburn, E. L. et al. [33], respectively. Due to the lack of availability of complete processed result data and the difficulty in accessing the raw datasets, the study by Meaburn, E. L et al., was not included in the current analysis. Additionally, we also searched for recent meta-analysis studies to compare the results we generated by processing the datasets available from GEO. Thusly, one meta-analysis study on data generated from brain samples and a mega-analysis data generated from blood samples of autistic subjects were selected [34,35] for the results comparison.

### 2.4. Nomenclature Mapping and Gene Family Association

Prior to enrichment analysis, mapping of entity names to their respective standard nomenclatures was performed for the protein and gene entities using the multi-symbol checker tool in the HGNC database. This standard nomenclature mapping ensures no protein or gene markers are missing during the enrichment analysis due to name mismatching.

### 2.5. Gene Set Enrichment Analysis, Cellular Process Mapping and Network Visualization

The software packages used for the functional enrichment analysis of the DEGs have been selected for their ease of use, free access, advanced features, extensive documentation and up-to-date databases. These tools include g:Profiler [36] (https://biit.cs.ut.ee/gprofiler/ accessed on 2 October 2020) and the ConsensusPathDB-human (CPDB—http://cpdb.molgen.mpg.de/ accessed on 2 October 2020) [37]. The HGNC gene symbol of the DEGs was used as the query in the g:GOST functional profiling interface in g:Profiler using Homo sapiens as the organism species, g:SCS threshold as the significance threshold and the user-defined *p*-value threshold as 0.05 in the advanced options. Similarly, the “over-representation analysis” feature was used for the enrichment analysis using the HGNC gene symbol as the gene identifier in the CPDB database. An interactive network (protein–protein, gene–protein and metabolite–protein) was framed by utilizing either the CPDB database using the induced network module analysis feature or the STRING web-based tool (v11.05) to evaluate the relationship among the DEGs from the datasets [38]. To attain a strong protein–protein interaction (PPI) network, we fixed the cutoff standard to a high confident interaction score ≥ 0.77 on the full STRING network. The PPI network extracted either from the STRING tool or the CPDB database was further subjected to detailed analysis by importing the network to Cytoscape software (v.3.9) (http://www.cytoscape.org/) [39]. For highly complex large PPI networks, cluster-based analysis using the Molecular Complex Detection plugin (MCODE) in Cytoscape software was performed to identify the clusters of densely connected molecular complexes [40] that could potentially align with the same biological goals as identified from the enrichment analysis results. To facilitate the biological interpretation as well as to visualize the functionally grouped Gene Ontology/pathway term networks and associated markers, Cytoscape plugins ClueGo and CluePedia were used [41,42]. ClueGO v2.5.8 and CluePedia v1.5.8 are Cytoscape plugins designed for functional enrichment analysis and visualization of biological networks. ClueGO creates functionally grouped annotation networks by integrating Gene Ontology (GO), KEGG, Reactome, and other pathway databases, allowing for an intuitive representation of gene functions. CluePedia extends ClueGO by enabling the integration of experimental data and additional molecular interactions, providing a deeper functional context to gene networks.

### 2.6. Gene Variant Association Analysis

Genetic risk variants for ASD traits, identified from GWAS, have been reported to show a statistically significant association with the ASD traits that are functionally enriched for genes involved in neurobiological and immune-related cellular processes [43]. However, linkage disequilibrium (LD) between nearby SNPs makes it difficult to identify the causal variants underlying the GWAS results. In the current study, for the gene-variant association analysis, we aimed to explore SNPs that map to glycogenes as a model example because of the biological importance of glycosylation PTM in autistic phenotypes [23]. To do this, we first identified all SNPs from the GWAS Catalog (https://www.ebi.ac.uk/gwas/ accessed on 2 October 2020) that are associated with ASD or autism disease (dated 15 January 2022). Out of the 712 unique list of SNPs identified from 38 studies (Supplementary Table S8), SNPs that were within 10 Kb of the start or end of any of the glycogenes were identified using a custom Perl script (query SNPs). For each of these query SNPs, we tested for LD between the SNP and a cis-eQTL for the corresponding target gene (glycogene) using LDexpress [44]. This analysis was based on the GTEx gene expression data [45] from brain and whole blood tissue samples and LD derived from the Chinese and European populations, because the majority of the autism-associated SNPs in the GWAS catalog were derived from studies in these populations. For the LD analysis, we used a window size of 100 kb and thresholds of *p* ≤ 0.05 and R^2^ ≥ 0.6.

## 3. Results

### 3.1. Functional Enrichment Analysis

**Figure 1:**
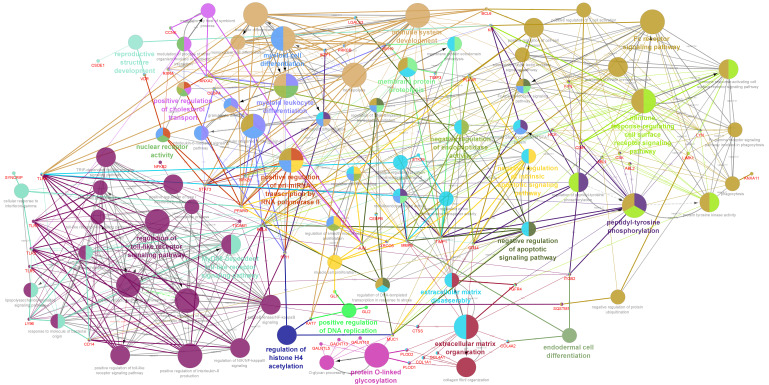
Gene ontology and pathway annotation network generated using the DEGs identified from the clustered PPI network analysis for the brain meta-analysis data [25]. The graph was generated using the via Cytoscape software (v.3.9). The DEGs used for the enrichment analysis are represented as the red-colored entity nodes that are interconnected with the GO process terms. The GO-Biological Process-EBI-Unirpot-GOA-ACAP-ARAP-13.05.2021 was selected for the ontologies/Pathways, and the evidence column was manually selected, including the “All_Experimental”; IGI; IPI; RCA; and TAS parameters. Pathways with *p*.value ≤ 0.05 were selected for the filtering criteria and the CluePedia Option of five genes per term was selected for the visualization threshold. The layout was manually arranged and analyzed after adding genes to the CluePedia network. All additional settings were retained with the default values.

Gene expression results from the autistic brain tissue samples published in the meta-analysis study by Forés-Martos J et al. [34] were subjected to targeted pathway enrichment analysis using prior-knowledge gene subsets. Our analysis results showed statistically significant (*p*.value ≤ 0.05; FDR ≤ 0.1) DEGs specific to the glycosylation pathways (64 genes), inflammatory response (372 genes), autism risk genes (132 genes) and ECM components (129 genes) (Supplementary Table S1). Pathway enrichment analysis revealed Glycosylphosphatidylinositol (GPI)-anchor biosynthesis, Glycosaminoglycan metabolism (chondroitin sulfate, dermatan sulfate and keratan sulfate), mucin type *O*-glycan biosynthesis and sialic acid metabolism as the most specific pathways associated with the glycogene subset. DEGs among the inflammatory gene subsets were represented by the Complement and coagulation cascades as well as multiple inflammatory signaling pathways (such as B-cell receptor signaling, NF-kappa B signaling, JAK-STAT signaling, TNF signaling, IL-17 signaling and the Toll-like receptor signaling). Among the Autism Risk genes, the synaptic signaling and transmission pathways, as well as neurotransmitter ones, were the most enriched pathways. Collagen Type IV Alpha 1 Chain (*COL4A1*), which encodes a major component of type IV collagen essential for basement membrane integrity, and *PLOD1*, which encodes lysyl hydroxylase 1 (LH1), an enzyme crucial for collagen cross-linking and structural stability. (Supplementary Table S2). The PPI network analysis using the 624 unique DEGs from the prior knowledge gene sets generated a complex interconnected network of ~1916 edges and 498 nodes. Cluster-based analysis of this network yielded 13 clusters with an average gene size of 5 (maximum size *n* = 19 genes and minimum size *n* = 2 genes (Figure 1)). The largest cluster (Cluster No:1; gene size *n* = 19, Supplementary Figure S2) formed among these DEGs generated an interconnected pathway module of immune response regulating cell surface receptor signaling pathway, majorly represented by the peptidyl-tyrosine phosphorylation and Fc receptor signaling pathways (Supplementary Table S2 and Figure 1). These pathways are well known to be a critical mediator of multiple signaling networks triggered by growth factors, cytokines, as well as the integrins and proteoglycans from the extracellular milieu that are known to be essential for neurite outgrowth and axon guidance [46,47]. The other larger clusters (Cluster No: 12 and 5; Supplementary Figure S2) majorly represented regulation of toll-like receptor signaling pathway and transcriptional regulators involved in myeloid leukocyte differentiation (Supplementary Table S2 and Figure 1). Other DEGs associated with the peptidase activity and proteolysis pathways formed pathway interconnections with the DEGs associated with the extracellular matrix organization and disassembly (Figure 1), which is a major source for extracellular signaling molecules vital for synaptic remodeling as well as regulating immune responses [48]. Similarly, the glycogene DEGs associated with the mucin type *O*-glycan biosynthesis were identified to form interconnected clusters with the proteases and peptidase family (Figure 1) that play a vital role in growth factor signaling, antigen presentation, matrix remodeling, as well as protein digestion.

**Figure 2:**
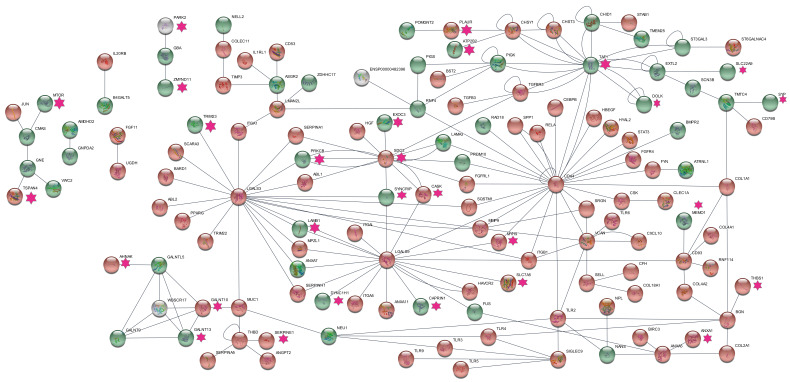
Subnetwork analysis of the DEGs centered on glycogenes extracted from the induced network model (CPDB database) generated for the prior knowledge gene subsets identified from the brain meta-analysis data [25]. The network is generated using the Cytoscape software (v.3.9), by manually selecting the interactions associated with the glycogene DEGs from the mother network file. Nodes were visualized using the “STRINGify network” feature and the fold change values for the DEGs were used to color the nodes. The red-colored nodes represent the upregulated genes, and the green-colored nodes represent the downregulated genes. The pink-colored star-shaped annotation represents the autism risk genes.

Detailed subnetwork analysis of this network to identify the biological significance of glycogenes was undertaken by retaining only the molecular network interactions centered on the glycogenes. The network revealed a highly connected neuro-inflammatory pathway linking the DEGs belonging to glycogenes, inflammatory response genes, and the autism risk genes (Figure 2). Galectins (*LGALS3*, *LGALS9*), Proteoglycans (*SDC2* (syndecan 2) and *CD44* (CD44 molecule (IN blood group)) and the transcription initiation factor *TAF1* (TATA-box binding protein associated factor 1) were identified as the major hub molecules in this network. Among the interacting proteins the autism risk specific genes that form the interconnected network with the glycogenes (especially the lectins and the proteoglycans) represented biological proteins that are majorly involved in cell–cell junctions, cell adhesion and migration, actin-cytoskeletal organization, vesicle transport and transcription factors that could play vital role in synaptic plasticity in neurons as well as cell proliferation and migration in multiple cell types (Figure 2; Supplementary Table S2).

**Figure 3:**
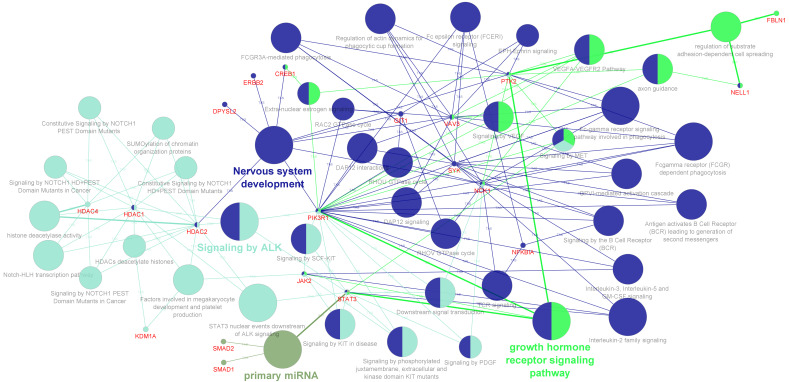
Gene ontology and pathway annotation network generated using the DEGs identified from the clustered PPI network analysis for the single-cell brain gene expression data [25]. The graph was generated using the ClueGo v2.5.8 + CluePedia v.1.5.8 plugin via the Cytoscape software (v.3.9). The DEGs used for the enrichment analysis are represented as the red-colored entity nodes that are interconnected with the GO process terms and the Reactome pathway terms selected for the analysis. The GO-Biological Process-EBI-Unirpot-GOA-ACAP-ARAP-13.05.2021 and the Reactome-Pathways-24.01.2022 were selected for the ontologies/Pathways. The evidence column was manually selected, including the “All_Experimental”, IGI, IPI, RCA, and TAS parameters. Pathways with *p*.value ≤ 0.05 were selected for the filtering criteria and the CluePedia Option of five genes per term was selected for the visualization threshold. The layout was manually arranged and analyzed after adding genes to the CluePedia network. All additional settings were retained with the default values.

In addition to the bulk gene expression study in the brain, we also performed a similar analysis with the single cell genomics study of cortical layers tissue from patients with autism, published by Velmeshev, D. et. al. [49]. Using prior knowledge gene-sets, specific inflammatory response genes and autism risk genes from this dataset were identified to be more enriched in the Layer 2/3 (L2/3) as well as Layer 4 excitatory neurons (L4), vasoactive intestinal polypeptide (VIP)–expressing interneurons (IN-VIP), microglia, endothelial cells, protoplasmic astrocytes and the parvalbumin interneurons (IN-PV) (Supplementary Table S3). As reported in the literature, the DEGs identified from the autism risk-specific genes were associated with the neurotransmitter release and synaptic vesicle pathway in the L2/3 excitatory neurons and IN-VIP interneurons (Supplementary Table S3). Similarly, the enrichment pathway analysis of the inflammatory gene set from the microglial cell type highlighted growth factor and interleukin signaling pathways, while the L4 excitatory neurons were enriched for the nuclear receptor transcription pathway and the sumoylation pathway with minimum gene representation (Supplementary Table S3). The whole DEGs across cell types were considered for the PPI network analysis, as the network generated from the CPDB database is agnostic of the tissue or cell types. Cluster analysis of this network yielded 5 clusters with the non-receptor tyrosine kinases and the signaling adaptor proteins forming the major connected cluster (Supplementary Figure S3). Gene ontology and pathway annotation-based network analysis of the DEGs from these clusters highlighted the role of non-receptor tyrosine kinases and the signaling adaptor proteins in diverse cellular signaling pathways, which play a vital role in neuronal development and plasticity [50] (Figure 3).

Even though not represented by the pathway annotation map, DEGs from the network clusters also incorporated key proteins involved in neuronal communication, development and plasticity such as *GPHIN* (gephyrin, molybdenum cofactor), *CAMK4* (Calcium/calmodulin-dependent protein kinase IV) and *CSDE1* (Cold Shock Domain Containing E1, an autism risk gene) [51,52,53] (Supplementary Figure S3). From the glycogene and ECM subsets, a very minimal amount of DEGs were identified across cell types, with endothelial cells having significant representation of differentially regulated ECM proteoglycans, which play a vital role in maintaining the brain micro-environment [54].

**Figure 4:**
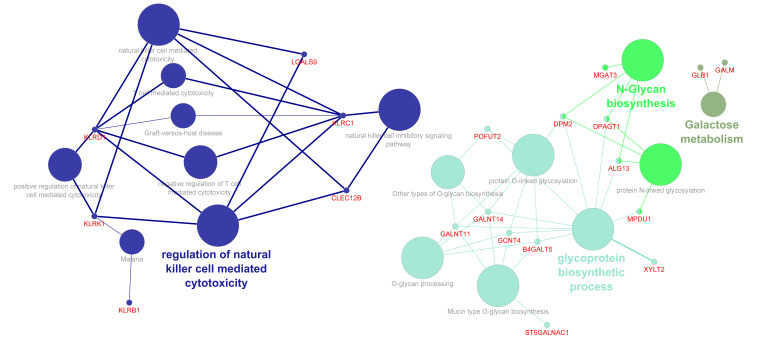
Gene ontology and pathway annotation network for the glycogene DEGs identified from the autistic blood samples [35]. The graph was generated using the ClueGo v2.5.8 + CluePedia v.1.5.8 plugin via the Cytoscape software (v.3.9). The DEGs used for the enrichment analysis are represented as the red colored entity nodes that are interconnected with the ontologies/pathway terms selected for the analysis. The ontologies GO-Biological Process-EBI-Unirpot-GOA-ACAP-ARAP-13.05.2021, the Reactome-Pathways-24.01.2022 and the WikiPathways_13.05.2021 were selected for the pathway enrichment analysis. The evidence column was manually selected, including the “All_Experimental”, IGI, IPI, RCA and TAS parameters. Pathways with *p*.value ≤ 0.05 were selected for the filtering criteria and the CluePedia Option of five genes per term was selected for the visualization threshold. The layout was manually arranged and analyzed after adding genes to the CluePedia network. The minimum gene representation in the GO Term/Pathway selection was relaxed to 1 to successfully map the genes to relevant process or ontology terms.

To compare the brain transcriptional and associated molecular pathway footprints from autistic individuals with those from the blood samples, we performed similar targeted enrichment analysis using previously published mega-analysis results from a blood transcriptomic comparison study in individuals with or without autism spectrum disorder [35]. As mentioned in this study, a relaxed FDR cut-off value q ≤ 0.05 was used to filter the DEGs for the enrichment analysis study. Among the prior-knowledge gene subsets, inflammatory response genes were the most represented (Supplementary Table S4). Functional enrichment analysis using the CPDB database yielded diverse cytokine and growth factor signaling pathways in the immune system, as well as the immunoregulatory interactions between lymphoid and non-Lymphoid cells (Supplementary Table S5) as the major pathways, which are also implicated in the regulation of growth factor and cytokine receptor-mediated signaling in hematopoiesis [55,56]. For the glycogene subsets, like the results obtained from the brain data, mucin type *O*-glycan biosynthesis and biosynthesis of the *N*-glycan precursor pathways (specifically, the formation of the dolichol-linked precursor) were identified (Supplementary Table S5; Figure 4) as the enrichment pathways. Whilst the differentially regulated lectins were associated with the pathways involved in Natural Killer cell-mediated cytotoxicity (Figure 4). A subnetwork focusing on the glycogenes was studied by manually extracting the reaction pathways from the large complex PPI network generated from the STRING database (Supplementary Figure S4). In this network, the differentially regulated glycoenzymes were found to form molecular complexes with proteins involved in cell cycle regulatory proteins, transcription factors, cell adhesion and actin-cytoskeleton proteins, which play a vital role in cell survival, migration and polarization. Similarly, the differentially regulated genes encoding lectin proteins from the *C*-type lectin-like domain (CLD) family were primarily associated with protein complexes involved in antigen presentation and interaction between lymphoid and non-lymphoid cells. Hence, it would be interesting to validate whether altered levels of hematopoietic cytokines and other hematopoietic developmental pathways are modified in the autistic subjects, which in turn might correlate with the autism spectrum disorder-related inflammation [57].

We further expand our research to additional gene expression datasets queried from the GEO database, especially those that are not covered by the above-mentioned meta-analysis and mega-analysis studies. In total, five blood tissue-based studies were identified that satisfied the filter criteria as mentioned in Section 2.1. Among the blood sample datasets, two datasets represented age-matched infants and toddlers, two for age-matched adults and one dataset for the children with a mean average age of 8.11 years (not an age-matched control) (Supplementary Table S6). Prior-knowledge gene set-based enrichment analysis of the blood transcriptomic data was applied for the adult and the infant blood sample datasets. Only DEGs that are either upregulated or downregulated by 1.5-fold values were considered for the analysis that meet the filtering criteria (Supplementary Table S6). Interestingly, data from the adult blood tissue contained a greater number of DEG representations when compared to that of the infants and toddlers. Due to a lack of sufficient sample size and age-matched controls, data from the children’s age category were not subjected to detailed analysis.

**Figure 5:**
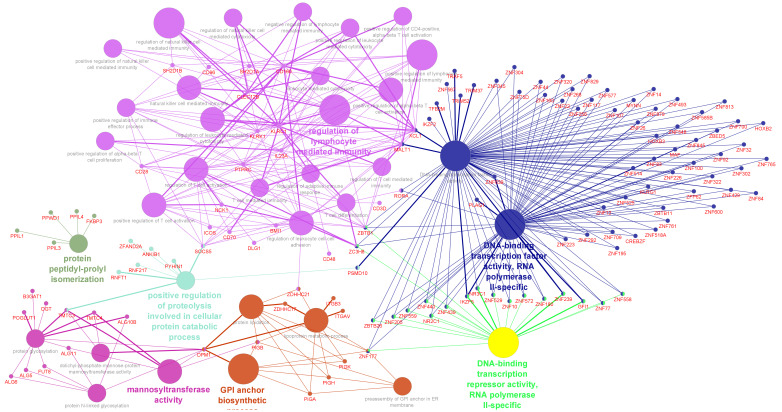
Gene ontology and pathway annotation network for the DEGs identified from the autistic adult blood samples [35]. The graph was generated using the ClueGo v2.5.8 + CluePedia v.1.5.8 plugin via the Cytoscape software (v.3.9). The DEGs used for the enrichment analysis are represented as the red colored entity nodes that are interconnected with the ontologies/pathway terms selected for the analysis. The ontologies GO-Biological Process-EBI-Unirpot-GOA-ACAP-ARAP-13.05.2021 were selected for the pathway enrichment analysis. By default, all parameters in the evidence column were selected for pathway identification. Pathways with *p*-value ≤ 0.05 were selected for the filtering criteria and the CluePedia Option of 10 genes per term was selected for the visualization threshold. The layout was manually arranged and analyzed after adding genes via the CluePedia application.

Even though the sample size was smaller, the gene expression pattern indicates that the glycogene DEGs belonging to the GPI-anchor biosynthesis pathway, as well as *N*-glycan biosynthesis, were more represented in the adult blood tissue. (Supplementary Table S7; Figure 5). However, no significant pathway enrichment results were obtained for the glycogene subsets from the infant blood tissue data. On the contrary, the PPI network analysis revealed interaction of specific glycogenes, proteoglycans and lectins with the T1CAM-dependent activation of the IR3/IR7pathway (Supplementary Figure S5), which is regarded as the early phase of extracellular signal-mediated activation of immune response [58]. Similarly, the glycogene subset focused analysis of the large PPI network from the adult blood tissue data conveys the association of these genes mainly with the transcription factors associated with immune response (Supplementary Figure S6). Collectively, the differential expression of transcription regulatory protein network involved in modulating diverse inflammatory responses and the glycogene network clusters associated with *N*-glycan biosynthesis and GPI-anchor biosynthesis pathways might indicate differential regulation of glycogenes in adult autistic blood samples that play vital role in blood-antigen biosynthesis and other post-translational modifications essential for the interaction between circulatory blood cells and endothelia as well as differentiation, maturation and activation of immune cells [59,60].

### 3.2. Linkage Disequilibrium Analysis of ASD-Associated SNPs in Glycogenes Using GWAS and eQTL Data

With the filter criteria applied, nine query variants were identified to be in Linkage Disequilibrium (LD) with 269 variants that mapped to 5 glycogenes (R^2^ ≥ 0.6; D′ ≥ 0.98) in the blood and brain tissues from the European and Chinese target population (Supplementary Table S9). Among these glycogenes, the Lectin Mannose-Binding 2 Like (LMAN2L) harbored the highest number of variants mapped in both blood and brain tissues (Supplementary Table S9). In contrast, Carbohydrate Sulfotransferase 1 (CHST1) and Polypeptide N-acetylgalactosaminyltransferase 10 (GALNT10) were found to contain variants uniquely mapped to blood and brain tissues, respectively (Supplementary Table S9).

Notably, glycosyltransferase 8 domain containing 1 (*GLT8D1*), which encodes a glycosyltransferase enzyme [61] for which the exact biological function is not yet characterized. However, in the Human Phenotype Ontology database, this gene has been linked to several human phenotypes, including Xerostomia (HP:0100756—Dryness of the mouth due to salivary gland dysfunction) [62], a condition that has been reported in some autistic individuals.

Similarly, the Neuraminidase 1 (*NEU1*) gene, associated with sialic acid metabolism—including polysialic acid synthesis—plays a critical role both in neuronal functions [63] and the modulation of immune responses [64].

Among the identified glycogenes, the polypeptide N-acetylgalacosaminyltransferase 10 gene (*GALNT10*) was identified to have the maximum number of variants mapped in the brain tissue (Supplementary Table S9). *GALNT10* has previously been implicated in autism development [65] (http://autism.mindspec.org/autdb/Welcome.do, accessed on 6 May 2022) and encodes a member of the GalNAc polypeptide *N*-acetylgalactosaminyltransferase family, which catalyzes the first step of mucin-type oligosaccharides biosynthesis [66]. Mucin-type oligosaccharides are evolutionarily conserved and play a vital role in gut microbe homeostasis, development and immune responses [67,68]. Thus, investigating whether alterations in *GALNT10* activity affect gut microbiota balance or immune dysregulation in individuals with ASD could offer important mechanistic insights.

A review of existing literature further supports the potential neurodevelopmental relevance of these glycogenes. *LMAN2L* (lectin, mannose binding 2 like), for instance, encodes a protein that belongs to the mannose-binding lectin family involved in processing and extracellular secretion of glycoproteins. Although not previously implicated in autism, genetic polymorphisms in *LMAN2L* have been significantly associated with attention-deficit/hyperactivity disorder (ADHD), bipolar disorder, schizophrenia, and intellectual disability [69,70]. *CHST1* encodes Carbohydrate Sulfotransferase 1 (C4ST1), an enzyme responsible for transferring sulfate groups to internal galactose residues, particularly in keratan sulfate proteoglycans. A knockdown study in rodent models demonstrated its role in the biosynthesis of major keratan sulfate proteoglycans, such as phosphacan, during brain development [71]. Interestingly, the gene expression results from the meta-analysis data revealed that both *LMAN2L* and *GALNT10* were upregulated and the *NEU1* gene was downregulated in the ASD brain samples when compared to that with the healthy controls (Table 1). These findings underscore the importance of experimental validation of these glycogenes to assess their gene expression and variant associations, which may reveal disruptions in glycosylation pathways relevant to ASD in both brain and blood samples.

Collectively, these findings provide important insights into the potential regulatory mechanisms linking ASD-associated genetic variation to glycogene function. The observed linkage disequilibrium between GWAS-identified SNPs and cis-eQTLs suggests that these variants may exert their effects through modulation of gene expression rather than direct protein-coding changes. In this context, the identified glycogenes—*LMAN2L*, *GALNT10*, *NEU1*, *CHST1*, and *GLT8D1*—converge on key biological processes including glycoprotein processing, sialic acid metabolism, and extracellular matrix modification, all of which are critical for neuronal development, synaptic function, and neuroimmune interactions. Disruption of these glycosylation pathways may therefore contribute to ASD pathophysiology by altering cell–cell communication, immune signaling, and gut–brain axis dynamics. Furthermore, the tissue-specific distribution of LD-associated variants, particularly those enriched in brain versus blood, suggests that both central and peripheral mechanisms may be involved in mediating these effects. Taken together, our results support a model in which ASD-associated variants influence glycosylation-related pathways through regulatory mechanisms, highlighting glycogenes as promising candidates for further functional validation in the context of ASD.

## 4. Discussion

One of the most fundamental PTMs in cellular biology that has a profound impact on genetic regulation and phenotypic traits is glycosylation [76,77,78]. Previous work has demonstrated that altered glycosylation patterns are associated with autistic traits [23]. Moreover, differential expression in the urine levels of acidic glycosaminoglycans has also been correlated with sleep disturbances and gastrointestinal symptoms in autistic subjects, a feature integral to the autistic phenotype [25]. Despite this evidence, there is sparse knowledge regarding the molecular mechanisms underlying the phenotypic correlation of glycosylation PTM with autistic traits. Hence, an integrated analysis method using multiple OMICs could be leveraged to unveil the molecular mechanisms, which is regarded as a powerful tool for identifying disease mechanisms [79]. In the current study, contrary to the traditional gene enrichment analysis method of finding general trends in the huge list of genes, a targeted approach using prior knowledge gene subsets was adopted. This enabled us to focus on the differential expression pattern of glycogenes and ECM glycoproteins in conjunction with the immune response and autism risk gene subsets from the blood and brain samples of autistic individuals. The biological pathway enrichment and PPI network analysis of the prior knowledge-based DEGs unfold a previously unexplored avenue of the importance of glycogenes in the autistic brain and blood tissues. We feel that the findings may be poised to have a high impact on future ASD research from a glycobiology perspective.

One of the noticeable findings from the current analysis is that the transcripts associated with immune responses are over-represented in both blood and brain tissue samples of autistic subjects. Increasing scientific evidence points towards immune dysregulation as a common underlying factor for multiple ASD related health complications. Multiple studies, leveraging both immunocytochemistry and functional imaging studies, have reported astrocytes and microglial activation in autistic brains to be correlated with the neuroinflammatory pathways in autistic brains [80,81,82,83]. Similarly, studies on autistic blood samples also explain notable differences in the immune response milieu, such as altered natural killer cell-related activity [84], cytokine profiles [85,86,87] and T-cell responses [88]. Despite the limitations in extrapolating transcriptomics data for glycan structure predictions, in the current study, functional enrichment and PPI network analysis were performed to correlate the altered glycosylation pathways with the immune response differences observed in autistic subjects.

In this regard, differential expression of glycogenes involved in mucin-type *O*-glycan biosynthesis in both autistic blood and brain tissue samples deserves special attention. It is evident from previous studies that mucin type *O*-glycan is vital for maintaining vascular integrity in specific tissues such as the brain and lymph node, as well as for lymphocyte trafficking [89]. Moreover, extensive *O*-glycosylation of human blood platelets, endothelial cells, and plasma proteins is reported to protect them from proteolytic processing [90]. Given the extensively reported evidence of altered platelet activity in autistic subjects [91], it will be interesting to study whether differential expressions of mucin type *O*-glycosylation might play a central role in the hemostatic system. Through specific interactions with glycan-binding proteins, altered expression of mucin-type *O*-glycans has been suggested to participate in regulating NK cell cytotoxicity as well as apoptosis in T-lymphocytes [92,93]. In addition to the *O*-glycosylation pathways, transcriptomic analysis revealed that the glycogene subsets belonging to GPI-anchor biosynthesis, glycosaminoglycan metabolism and sialic acid metabolism are also differentially regulated in autistic subjects. Even previous research on copy number variants has also correlated a few glycogenes belonging to similar pathways in autistic individuals but without clearly establishing whether it is a cause or effect relationship [23]. However, these pathways are crucial in the synthesis and organization of neuronal extracellular glycocalyx and other glycoconjugate structures in the brain that are essential for neuronal development, survival, synaptic transmission and plasticity [94,95,96]. Moreover, results from the PPI network analysis indicate that the protein molecular complexes that are found to be interacting with the glycogene DEGs are critical for maintaining core cellular functions such as growth factor signaling, antigen presentation, matrix remodeling and protein digestion. Similarly, it is observed that the molecular complexes formed by the differentially regulated glycan-binding proteins (Galectins, C-type lectins) and proteoglycans play a vital role in cell–cell junctions, cell adhesion and migration, actin-cytoskeletal organization, vesicle transport and transcription regulatory factors. Collectively, the enrichment pathway and protein association network analysis of differentially regulated glycogenes identified in this study could implicate a potential role in core biological functions that are reported to be dysregulated in autistic subjects, such as neuro-inflammatory and synaptic remodeling processes [97,98] as well as other systemic immunological abnormalities [99].

To further substantiate the biological relevance of the identified glycogene signatures and variant associations, targeted experimental validation strategies are warranted. Proteomic and, more specifically, glycoproteomic profiling of brain, blood, or cerebrospinal fluid (CSF) samples from ASD individuals could provide direct evidence of altered glycosylation patterns, including site-specific glycan modifications and structural changes that cannot be inferred from transcriptomic data alone. Such approaches would enable validation of whether the observed transcriptional alterations translate into functional changes at the protein and glycan levels. In parallel, functional assays using neuronal or microglial model systems—such as induced pluripotent stem cell (iPSC)-derived neurons or microglia—could be employed to investigate the impact of perturbing key glycogenes (e.g., *GALNT10*, *NEU1*, *LMAN2L*) on synaptic function, immune signaling, and cell–cell interactions. Furthermore, evaluating the diagnostic potential of these glycogene signatures through biomarker studies in peripheral blood or CSF may help establish clinically relevant readouts associated with ASD-related glycosylation and immune dysregulation. Collectively, these integrative validation approaches will be essential to translate the current findings into mechanistic insights and potential therapeutic or diagnostic applications.

The study of glycosylation changes in neurological disorders is gaining momentum owing to the significant advancement being made in the field of glyco-based therapeutics as a strategy to modulate immune responses [100]. Hence, characterizing alterations in glycan structures as well as glycan binding proteins might reveal new avenues for therapeutic intervention as well as diagnostic markers for autism associated health complications, taking into account the heterogeneity and subtypes within ASD. Current analysis focusing on the results generated from the meta-analysis and mega-analysis studies has enabled us to derive transcriptomics patterns for the glycogenes in a broad tissue-specific manner. However, the lack of sufficient sample size in an age-matched, gender-specific, cell-specific and family history background limits the statistical significance as well as the biological functionality of identified glycosylation pathways stemming from extrapolated glycogene DEGs. Furthermore, the gene variant association analysis study also enabled us to identify variants that can potentially modulate the expression of key glycan genes whose biological function correlates with some of the autistic traits, as well as play a vital role in brain development, immune response and gut-microbe homeostasis. Hence, it is paramount to have an adequate study considering the above-mentioned parameters, as well as the heterogeneity and subtypes within ASD subjects, to identify the functional role of glycogenes in altered brain and systemic immune dysregulation observed in this population.

Despite the novel insights offered by our integrative transcriptomic and network analysis framework, several limitations should be considered when interpreting the results. Firstly, the gene expression datasets used in this study were retrieved from publicly available repositories such as GEO, meta-analysis [34], and mega-analysis [35], and were generated across different platforms, populations, and experimental conditions. This heterogeneity may introduce batch effects and limit direct comparability between datasets.

Secondly, while our methodology incorporates blood and brain transcriptomic data, most analyses were based on bulk RNA sequencing, which may obscure cell–type–specific expression patterns. Although we addressed this partially by analyzing single-cell transcriptomic data from Velmeshev et al. [49], the lack of matching age, sex, and phenotypic metadata limits the generalizability of these findings.

Third, the gene sets representing glycogenes, inflammatory markers, and autism risk genes were compiled from multiple knowledge bases, which, although robust, may still be incomplete or biased toward better-studied genes. Likewise, our focus on transcriptional changes does not account for post-transcriptional regulation, protein-level expression, or actual glycan structure modifications, which are critical to validating functional glycosylation changes.

Importantly, while we incorporated ASD-associated SNP data from the GWAS catalog (dated 15 January 2022), the eQTL and LD analyses relied on data derived primarily from European and Chinese populations. This population bias may not capture variant effects in underrepresented ethnic groups, limiting the universality of variant-glycogene associations.

Lastly, due to limitations in sample availability, some age groups—particularly infants and toddlers—were underrepresented or excluded from detailed pathway analyses. Furthermore, the absence of matched control data for certain groups reduces the statistical power to detect subtle, yet biologically meaningful, changes.

Future studies incorporating longitudinal, multi-omic, and glycomics datasets, with larger sample sizes and better metadata standardization, will be essential to validate and extend these findings.

## 5. Conclusions

**Figure 6:**
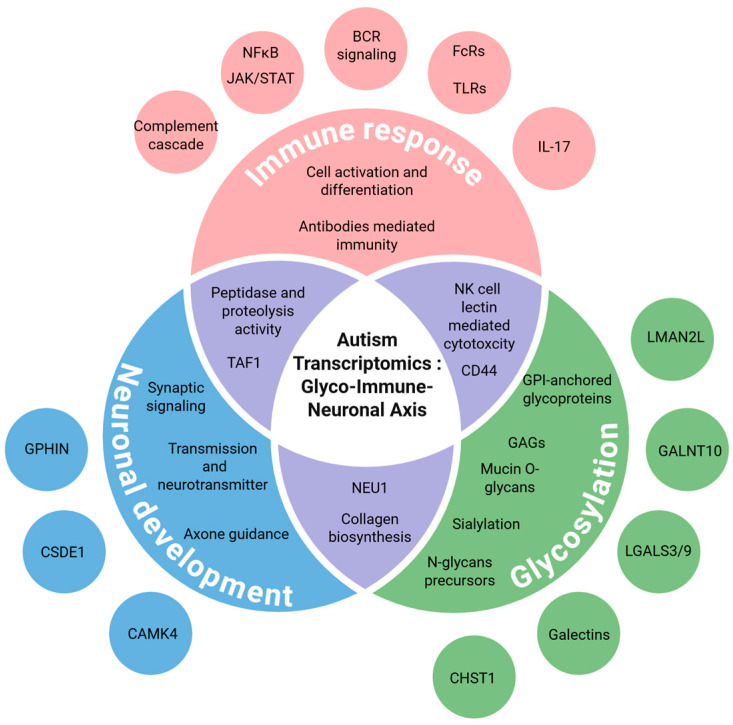
The glyco-immune axis in autism: an integrative representation of molecular pathways linking glycosylation, immune response, and neuronal development. The diagram illustrates the interconnected roles of glycosylation, immune response, and neuronal development pathways in ASD. Key findings highlight transcriptional dysregulation in glycan biosynthesis (e.g., mucin O-glycans, GPI-anchors), neuroinflammatory signaling (e.g., IL-17, JAK/STAT), and synaptic processes. Central glyco-immune-neuronal interactions are represented by shared gene functions and pathway overlap, suggesting an integrated molecular framework contributing to ASD pathophysiology. Created in BioRender. Morel, M. (2026) https://BioRender.com/cozt6a8 accessed on 2 October 2020.

This study highlights a convergent network of dysregulated glycosylation, immune response, and neuronal development pathways in ASD, forming a distinct glyco-immune axis (Figure 6). By integrating transcriptomic data across tissues and datasets, we demonstrate that specific glycogenes—including those involved in mucin-type *O*-glycans, sialylation, and GPI-anchor biosynthesis—interact functionally with immune signaling and synaptic pathways relevant to ASD pathophysiology.

The findings underscore glycosylation as a central and underexplored regulatory layer linking neuroinflammation and synaptic dysfunction in autism. This framework offers a basis for future studies to investigate glycan-mediated mechanisms and their potential as diagnostic or therapeutic targets in ASD.

## Figures and Tables

**Table 1:** The table presents previously published scientific literature evidence for the potential association of identified risk variants with the autism phenotype. Both direct and indirect evidence supporting the association are represented, wherein the indirect evidence refers to the literature that mentions the name of the gene and its potential association with the autistic phenotype. The up and down arrows under the brain samples column represent the gene expression pattern identified from the meta-analysis reports selected for the current study. No significant expression patterns were observed for these genes from the meta-analysis reports for the blood samples.

Glycogenes	Specific Pathway	Direct Evidence	Indirect Evidence	Brain Samples
Glycosyltransferase 8 domain containing 1 (GLT8D1)	Not yet characterized	[61]	[72,73]	na
Polypeptide N-acetylgalactosaminyltransferase 10 (GALNT10)	Mucin-type oligosaccharides	[43]		↑
Lectin mannose binding 2 Like (LMAN2L)	Processing and extracellular secretion of glycoproteins		[69,74]	↑
Neuraminidase 1 (NEU1)	Sialic acid biosynthesis		[75]	↓

## Data Availability

1. The differentially expressed genes, identified from the full ASD differential gene expression meta-analysis results of brain samples published by Forés-Martos, J. et al. is available from the following published article [34]. 2. The differentially expressed genes, identified from the full ASD differential gene expression mega-analysis results of blood samples published by Tylee DS. et al. is available from the following published article [35]. 3. The gene expression datasets analyzed during the current study are available in the GEO repository under the following GEO accession numbers GSE18123; GSE26415; GSE89594; GSE42133. 4. All data generated or analyzed during this study are included in this published article and its Supplementary Information Files.

## Supplementary Materials

The following supporting information can be downloaded at: https://www.mdpi.com/article/10.3390/genes17040486/s1, Figure S1: Overview of the workflow adopted for identifying altered glycosylation pathway associated with the dysregulated immune response in autistic subjects. The methodology employs analysing published datasets by leveraging open-source bioinformatics softwares and data analysis tools. Figure S2: Clustered PPI network generated using the cytoscape software (v.3.9), using MCODE clustering by selecting the Degree cut-off and K-Core parameter as 2 and with the maximum depth as 100 for the algorithm to search in the molecular complex prediction step. The graph displays only the clusters and the non-connected nodes were avoided via manual arrangement. Nodes were visualized using the “STRINGify network” feature and the fold change values for the DEGs were used to colour the nodes. The red colour nodes represent the upregulated genes and the green coloured nodes represent the downregulated genes. Cluster analysis was performed on the full network generated from CPDB database using the induced network modules by inputting the DEGs identified for the prior knowledge gene subsets (*representing inflammatory response genes*, *glycogenes*, *ECM genes and the Autism risk genes*) and setting the z-score threshold value of 20. Figure S3: Clustered PPI network generated using the cytoscape software (v.3.9), using MCODE clustering by selecting the Degree cut-off and K-Core parameter as 2 and with the maximum depth as 100 for the algorithm to search in the molecular complex prediction step. The graph displays only the clusters and the non-connected nodes were avoided by manual arrangement. Nodes were numbered according to the cluster they belong to, based on the MCODE clustering. Visualization of the nodes were performed using the “STRINGify network” feature and the fold change values for the DEGs were used to colour the nodes. The red colour nodes represent the upregulated genes and the green coloured nodes represent the downregulated genes. Cluster analysis was performed on the full network generated from CPDB database using the induced network modules by inputting the DEGs identified for the prior knowledge gene subsets (*representing inflammatory response genes*, *glycogenes*, *ECM genes and the Autism risk genes*) and setting the z-score threshold value of 20. Figure S4: Subnetwork analysis of the DEGs centered on glycogenes extracted from the PPI network from STRING database generated for the prior knowledge gene subsets identified from the blood mega-analysis data [35]. The network is generated using the cytoscape software (v.3.9), by manually selecting the interactions associated with the glycogene DEGs from the mother network file. Nodes were visualized using the “STRINGify network” feature and the fold change values for the DEGs were used to colour the nodes. The red colour nodes represent the upregulated genes and the green coloured nodes represent the downregulated genes. Figure S5: PPI network visualized using the cytoscape software (v.3.9), using DEGs identified for the prior knowledge gene subsets (*representing inflammatory response genes*, *glycogenes, ECM genes and the Autism risk genes*) from the autistic infant blood tissue transcriptomics data. The network is generated using the induced network module feature in CPDB database by inputting the DEGs. Visualization of the nodes were performed using the “STRINGify network” feature in Cytoscape and the nodes were manually arranged. Figure S6: Subnetwork analysis of the DEGs centered on glycogenes extracted from the induced network model (CPDB database) generated for the prior knowledge gene subsets identified from the autistic adult blood tissue transcriptomics data. The network is generated using the cytoscape software (v.3.9), by manually selecting the interactions associated with the glycogene DEGs from the mother network file. Nodes were visualized using the “STRINGify network” feature. Table S1: Statistically significant DEGs identified from the full ASD differential gene expression meta-analysis results of brain samples published by Forés-Martos, J. et al. based on the prior knowledge gene sets. Table S2: Functional Enrichment analysis of the DEGs identified based on prior-knowledge gene subsets from the full ASD differential gene expression meta-analysis results of brain samples published by Forés-Martos, J. et al. Table S3: Enrichment analysis using prior-knowledge gene sets for the single cell data obtained from the sutdy published by Velmeshev, D. et. al. Table S4: Statistically significant DEGs identified from the full ASD differential gene expression mega-analysis results of blood samples published by Tylee DS. et al. based on the prior knowledge gene sets. Table S5: Enrichment analysis results for the statistically significant DEGs identified from the full ASD differential gene expression mega-analysis results of blood samples published by Tylee DS. et al. based on the prior knowledge gene sets. Table S6: Prior-knowledge gene set-based enrichment analysis of the blood transcriptomic data for the adult and the infant blood sample datasets. Table S7: Enrichment Results for the Prior Knowledge Geneset based DEGs identified from the additional blood tissue samples. Table S8: SNPs_Autism_GWAS_Catalog (Dated: 15 January 2022). Table S9: Linkage Disequilibrium Analysis of ASD-Associated SNPs in Glycogenes Using GWAS and eQTL Data.

## Abbreviations

The following abbreviations are used in this manuscript:

ADHD	Attention-Deficit/Hyperactivity Disorder
ASD	Autism Spectrum Disorder
CDG	Congenital Disorders of Glycosylation
CNV	Copy Number Variations
DEG	Differentially Expressed Genes
DSM-5	Diagnostic and Statistical Manual of Mental Disorders
ECM	Extracellular Matrix
eQTLs	expression Quantitative Trait Locus
GEO	Gene Expression Omnibus
GPI	Glycosylphosphatidylinositol
GWAS	Genome-Wide Association Studies
HGNC	HUGO Gene Nomenclature Committee
ICD	International Classification of Diseases
LD	Linkage Disequilibrium
MDD	Major Depressive Disorder
MCODE	Molecular Complex Detection
PTM	Post-Translational Modifications
QoL	Quality of Life
RNA	Ribonucleic Acid
SFARI	Simons Foundation Autism Research Initiative
SNP	Single Nucleotide Polymorphism
