# Supplementary material for: An Integrative Network Analysis Framework for Identifying Altered Glycosylation Pathways Associated with Autism Spectrum Disorder
Source: Genes (Basel). 2026 Apr 19;17(4):486. doi: 10.3390/genes17040486 (PMC13115569; doi:10.3390/genes17040486)
Supplement: Supplementary file 1 [file genes-17-00486-s001.zip › Supplementary Figures (1).pdf]

Supplementary Figures

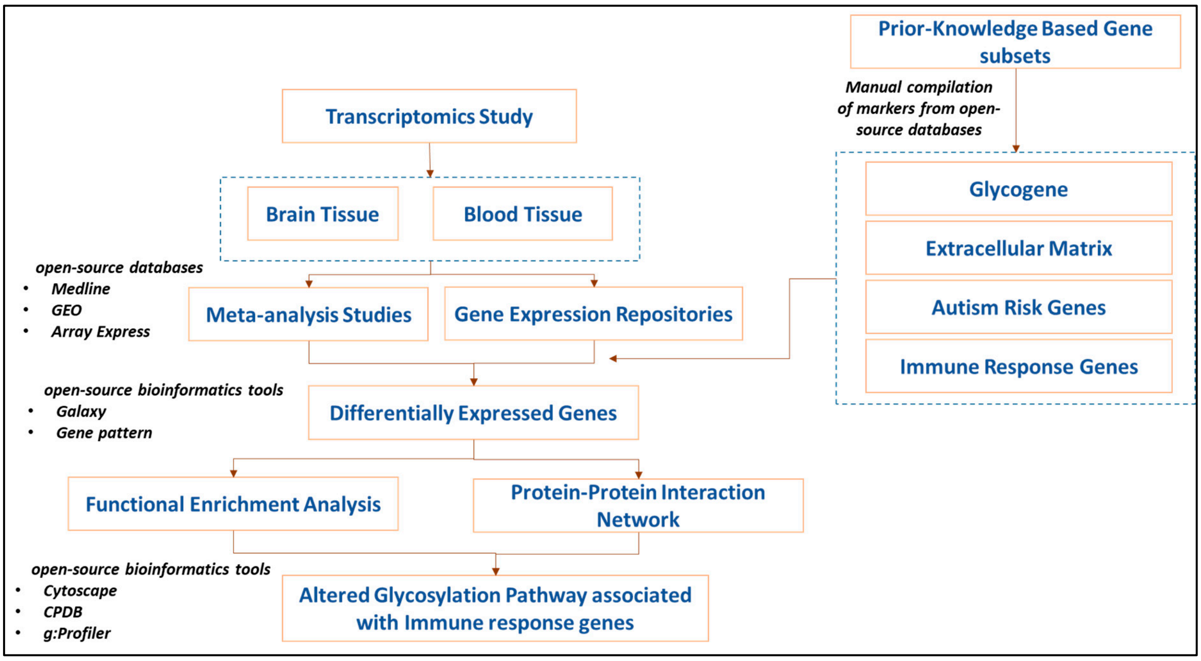

Supplementary Figure S1: Overview of the workflow adopted for identifying altered glycosylation pathway associated with the dysregulated immune response in autistic subjects. The methodology employs analysing published datasets by leveraging open-source bioinformatics softwares and data analysis tools.

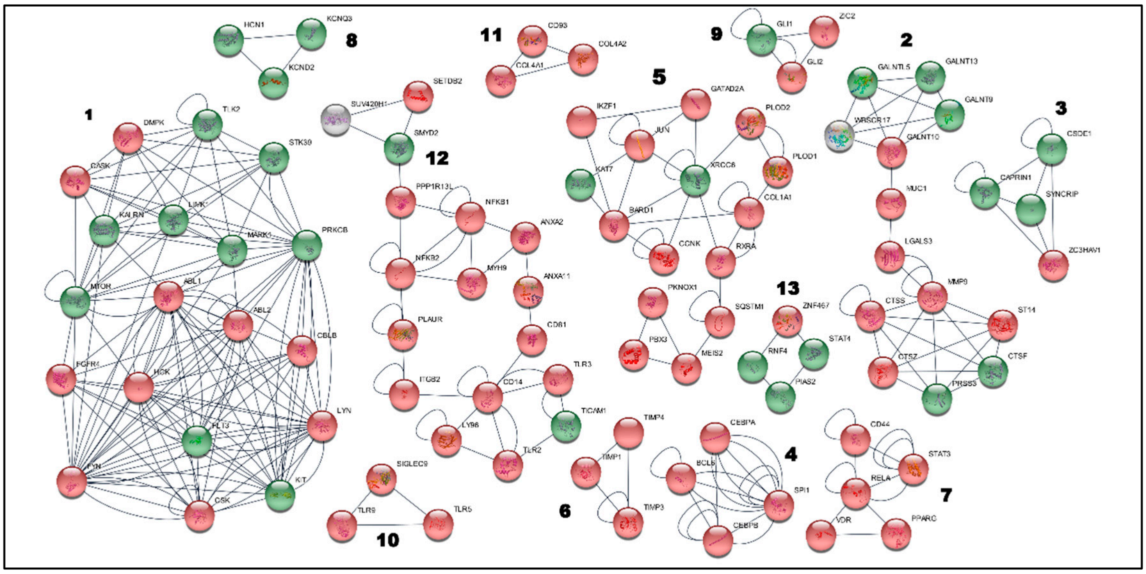

Supplementary Figure S2: Clustered PPI network generated using the cytoscape software (v.3.9), using MCODE clustering by selecting the Degree cut-off and K-Core parameter as 2 and with the maximum depth as 100 for the algorithm to search in the molecular complex prediction step. The graph displays only the clusters and the non-connected nodes were avoided via manual arrangement. Nodes were visualized using the "STRINGify network" feature and the fold change values for the DEGs were used to colour the nodes. The red colour nodes represent the upregulated genes and the green coloured nodes represent the downregulated genes. Cluster analysis was performed on the full

network generated from CPDB database using the induced network modules by inputting the DEGs identified for the prior knowledge gene subsets (*representing inflammatory response genes, glycogenes, ECM genes and the Autism risk genes*) and setting the z-score threshold value of 20.

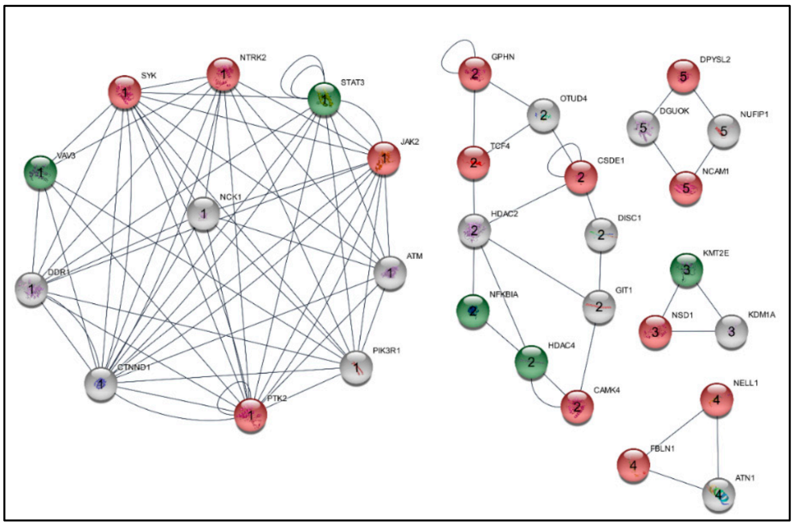

Supplementary Figure S3: Clustered PPI network generated using the cytoscape software (v.3.9), using MCODE clustering by selecting the Degree cut-off and K-Core parameter as 2 and with the maximum depth as 100 for the algorithm to search in the molecular complex prediction step. The graph displays only the clusters and the non-connected nodes were avoided by manual arrangement. Nodes were numbered according to the cluster they belong to, based on the MCODE clustering. Visualization of the nodes were performed using the "STRINGify network" feature and the fold change values for the DEGs were used to colour the nodes. The red colour nodes represent the upregulated genes and the green coloured nodes represent the downregulated genes. Cluster analysis was performed on the full network generated from CPDB database using the induced network modules by inputting the DEGs identified for the prior knowledge gene subsets (*representing inflammatory response genes, glycogenes, ECM genes and the Autism risk genes*) and setting the z-score threshold value of 20.

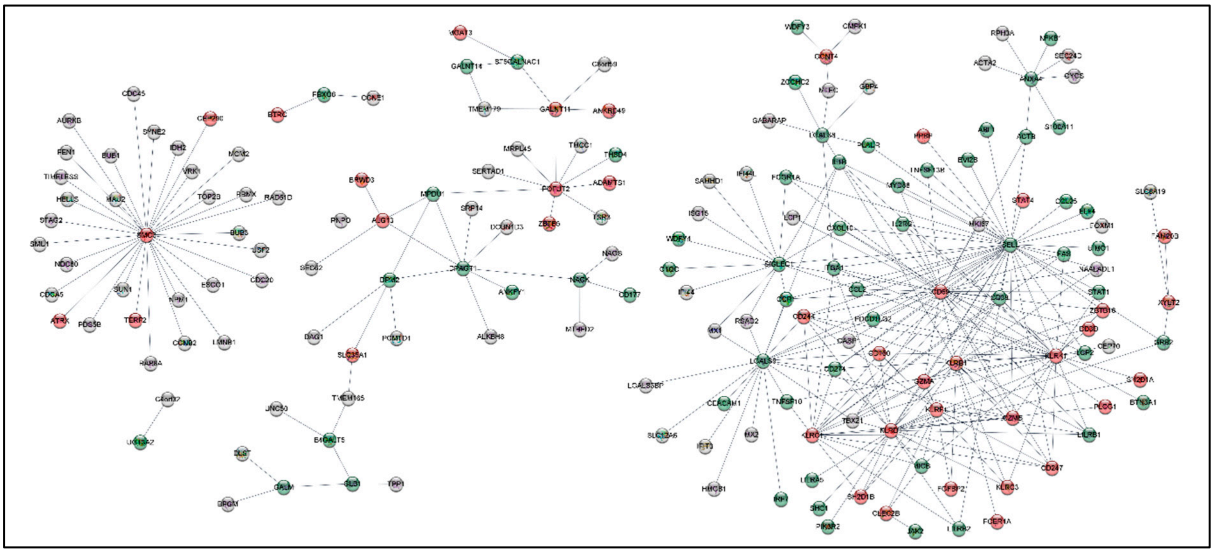

Supplementary Figure S4: Subnetwork analysis of the DEGs centered on glycogenes extracted from the PPI network from STRING database generated for the prior knowledge gene subsets identified from the blood mega-analysis data [35]. The network is generated using the cytoscape software (v.3.9), by manually selecting the interactions associated with the glycogene DEGs from the mother network file. Nodes were visualized using the "STRINGify network" feature and the fold change values for the DEGs were used to colour the nodes. The red colour nodes represent the upregulated genes and the green coloured nodes represent the downregulated genes.

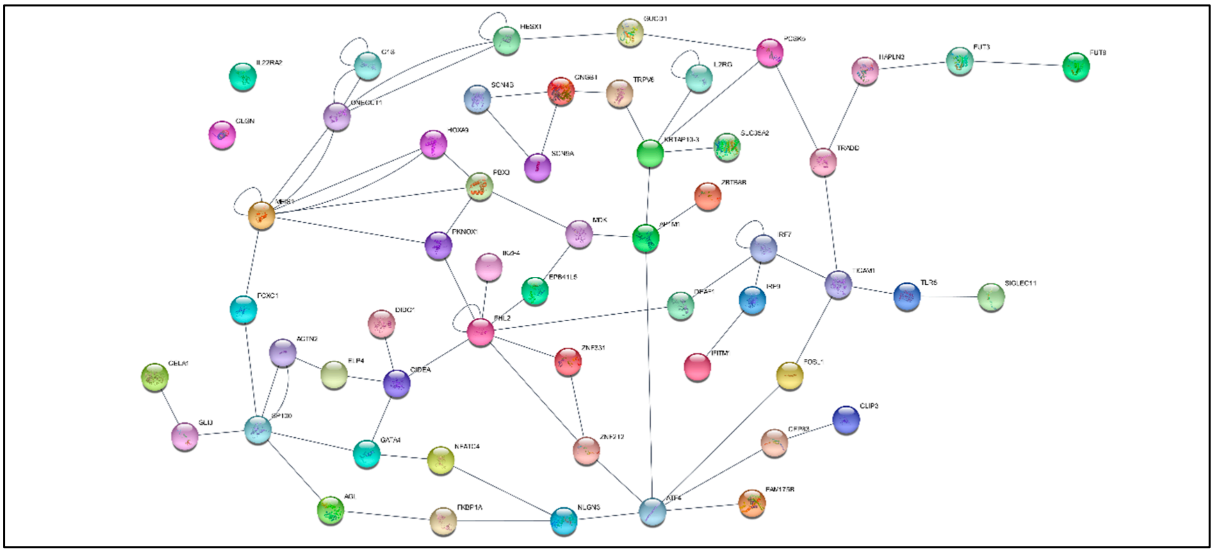

Supplementary Figure S5: PPI network visualized using the cytoscape software (v.3.9), using DEGs identified for the prior knowledge gene subsets (*representing inflammatory response genes, glycogenes, ECM genes and the Autism risk genes*) from the autistic infant blood tissue transcriptomics data. The network is generated using the induced network module feature in CPDB database by inputting the DEGs. Visualization of the nodes were performed using the "STRINGify network" feature in Cytoscape and the nodes were manually arranged.

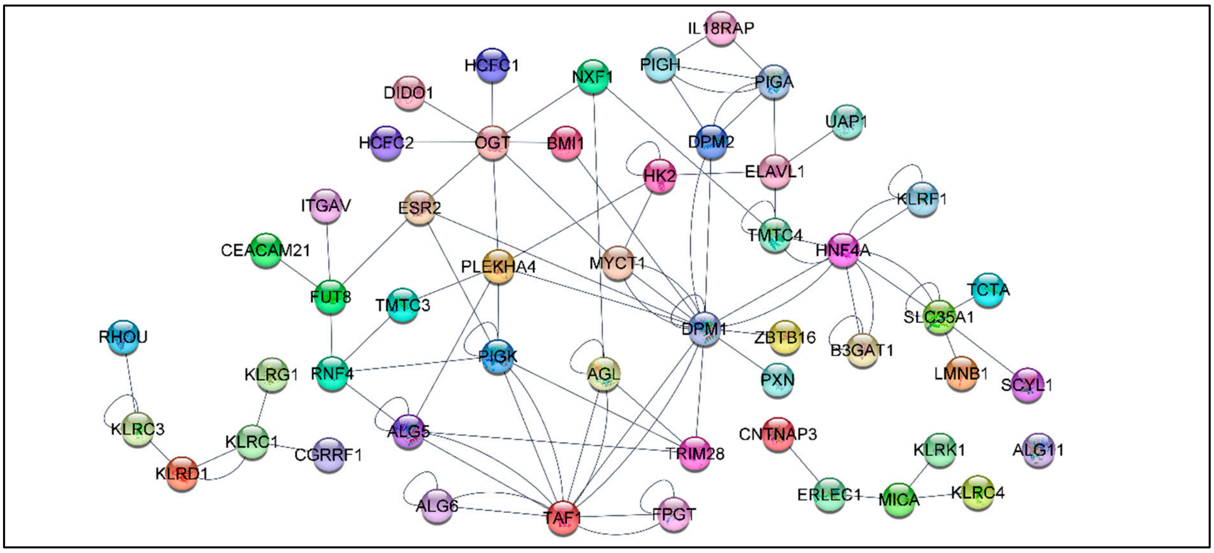

Supplementary Figure S6: Subnetwork analysis of the DEGs centered on glycogenes extracted from the induced network model (CPDB database) generated for the prior knowledge gene subsets identified from the autistic adult blood tissue transcriptomics data. The network is generated using the cytoscape software (v.3.9), by manually selecting the interactions associated with the glycogene DEGs from the mother network file. Nodes were visualized using the "STRINGify network" feature.
